# Cytarabine-Induced Encephalitis

**DOI:** 10.5334/jbsr.2492

**Published:** 2021-09-16

**Authors:** Christine Lenfant, Nathalie Greiner, Thierry Duprez

**Affiliations:** 1 Cliniques universitaires Saint-Luc, BE

**Keywords:** cytarabine, cytosar, cerebellar toxicity, neurotoxicity, cerebellar encephalitis

## Abstract

**Teaching Point**: Central nervous system adverse effects of cytarabine treatment include aseptic meningitis, myelopathy, and more rarely, encephalopathy, seizures, and cerebellar dysfunction. This case illustrates a cytarabine-induced encephalitis with predominant cerebellar involvement.

## Case Study

**Figure 1:**
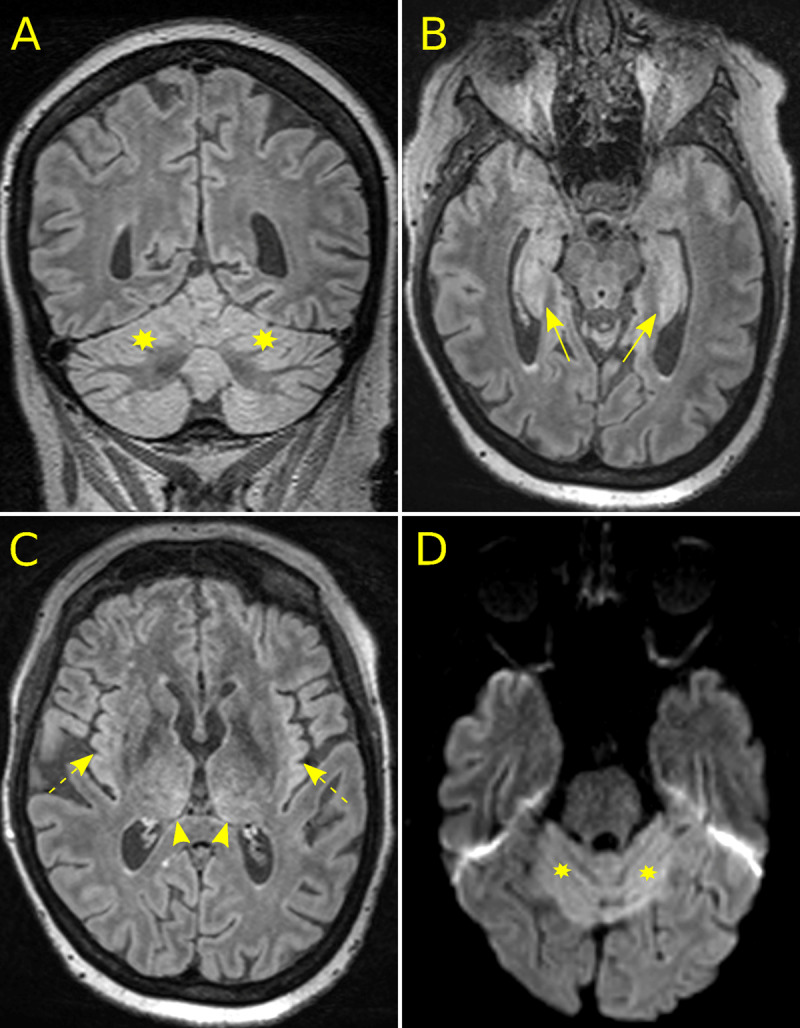


A 57-year-old woman with acute myeloid leukemia (AML) underwent induction followed by consolidation treatment protocol combining intravenous daunorubicine (60 mg/m^2^/day for 3 days of induction and 45 mg/m^2^/day for three days of consolidation) and high-dose cytarabine (200 mg/m^2^ /day for seven days of induction and 600 mg/m^2^/day for six days of consolidation). Dose was adapted to the decrease in renal function during the consolidation period. One week after the consolidation injections, she suffered from sudden onset of gait ataxia, dysphagia, and dysarthria. Cerebral computed tomography (CT) and CT angiography (not shown) excluded acute ischemic stroke. Magnetic resonance (MR) was performed under general anesthesia because of irrepressible claustrophobia (***Figure 1***). Symmetrically increased signal intensity was observed on fluid-attenuated inversion recovery (FLAIR) images within the cortex of the cerebellum (A, asterisks), the mesial temporal areas (B, arrows) and at a lesser degree, in the insular cortex (C, dotted arrows) and the posteromedial thalamic nuclei (C, arrow heads). Diffusion-weighted images demonstrated both increased signal intensity (D, asterisks) and reduced apparent diffusion coefficient values (not shown) within damaged areas. No blood-brain-barrier disruption was observed on post-contrast T1-weighted (not shown). After review of the literature, a toxic cerebellar encephalitis due to the administration of high-doses of cytarabine in a patient with progressively worsening renal function was hypothesized, and then confirmed after the nearly complete resolution of the cerebellar syndrome after withdrawal of cytarabine (only remained a slight dysarthria). Follow-up MRI was not performed as general anesthesia should have been inappropriately applied to a patient with ongoing neurological recovery.

## Comment

Cerebellar syndrome due to high-dose cytarabine treatment is a well-known clinical entity with few radiological documentation. The perfect match between clinical and MR features at acute phase together with the major clinical recovery after withdrawal of the presumptively causative drug strongly suggested that cytarabine had been responsible for the clinical/radiological features. Worsening renal function throughout the treatment course had obviously increased the risk for toxic side effects of the drug. However, subsidence of the radiological signs paralleling the clinical recovery could not be demonstrated as follow-up MRI was not feasible in ethical conditions. Nevertheless, sufficient evidence seemed to emerge from this observation to definitely include the cytarabine in the group of antimitotic drugs responsible for reversible acute cerebellar toxicity (REACT) syndrome, which has already done for other drugs such as the 5-fluorouracile [1]. Additional mesiotemporal, insular, and thalamic involvement in the condition has remained until now an undescribed feature of cytarabine-related brain toxicity since radiological reports in the condition are missing.
